# Supplementary material for: Splice-Junction-Based Mapping of Alternative Isoforms in the Human Proteome
Source: Cell Rep. Author manuscript; Available in PMC 2020 Jan 15. (PMC6961840; doi:10.1016/j.celrep.2019.11.026)
Supplement: 2 [file NIHMS1546469-supplement-2.zip › DF1/PXD009737/TestisGluC/TestisGluC_20_CDADC1_KKLRLGIKE.pdf]

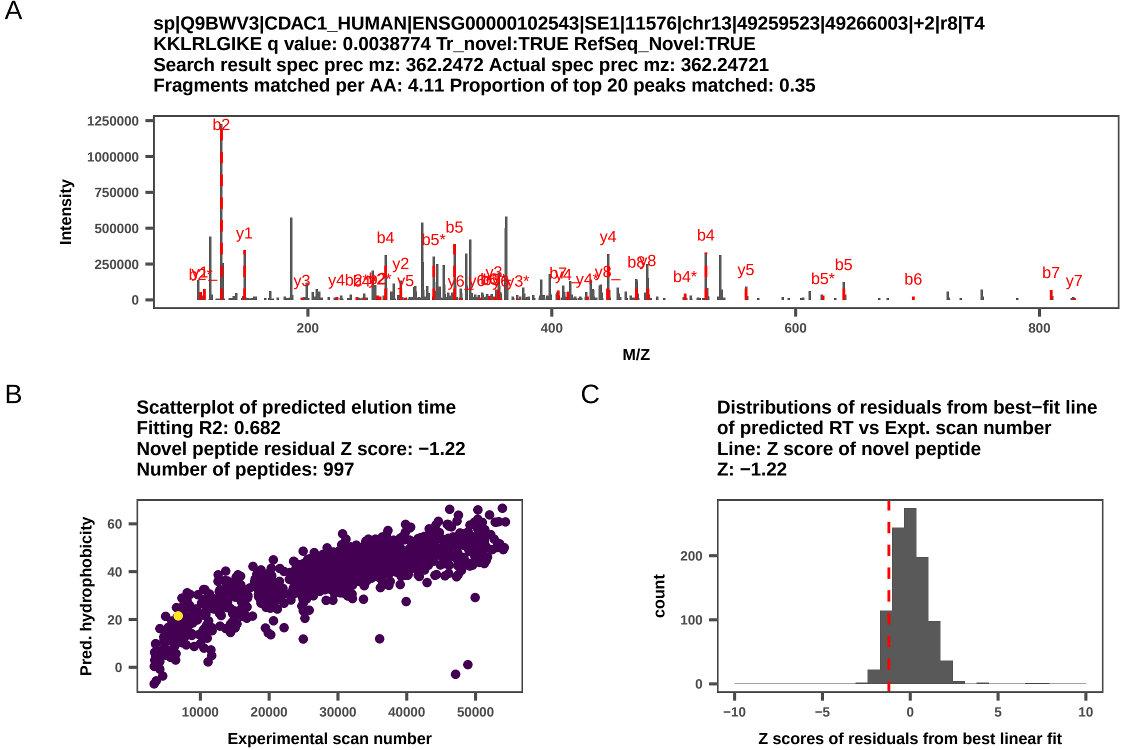
A
sp|Q9BWV3|CDAC1_HUMAN|ENSG00000102543|SE1|11576|chr13|49259523|49266003|+2|r8|T4
KKLRLGIKE q value: 0.0038774 Tr_novel:TRUE RefSeq_Novel:TRUE
Search result spec prec mz: 362.2472 Actual spec prec mz: 362.24721
Fragments matched per AA: 4.11 Proportion of top 20 peaks matched: 0.35
Intensity
1250000
1000000
750000
500000
250000
0
M/Z
200
400
600
800
b2
y1
b4
y2
b5*
b5
y3
y5
y6
y4
y8
b4
y5
b4*
b5*
b5
b6
b7
y7
B
Scatterplot of predicted elution time
Fitting R2: 0.682
Novel peptide residual Z score: −1.22
Number of peptides: 997
Pred. hydrophobicity
Experimental scan number
10000
20000
30000
40000
50000
0
20
40
60
C
Distributions of residuals from best−fit line
of predicted RT vs Expt. scan number
Line: Z score of novel peptide
Z: −1.22
count
Z scores of residuals from best linear fit
−10
−5
0
5
10
0
100
200
